# Photocrosslinked dual-network hydrogel for sutureless corneal stromal lenticule lmplantation

**DOI:** 10.3389/fbioe.2026.1764867

**Published:** 2026-02-23

**Authors:** Xianglong Yi, Yue Song, Liqun Chen, Riye Su, Bo Liu, Xiaohui Tang, Qing Wei, Yingbo Wang, Wenbo Cheng

**Affiliations:** 1 Department of Ophthalmology, The First Affiliated Hospital, Xinjiang Medical University, Urumqi, China; 2 College of Chemical Engineering, Xinjiang Normal University, Urumqi, China; 3 The First Affiliated Hospital of Xinjiang Medical University, Urumqi, China

**Keywords:** corneal stromal lenticule, hydrogel, sutureless corneal transplantation, tissue engineering, wound healing

## Abstract

Corneal stromal lenticules obtained through small incision lenticule extraction (SMILE) procedures offer a valuable graft material for therapeutic applications. Current clinical utilization faces challenges due to intrinsic thinness (<140 μm) and restricted dimensions (generally around 6.6 mm). This study introduced a novel approach to enable the construction of customizable corneal grafts by stacking lenticules, achieving specific thickness and diameter for diverse corneal defects, using photo-crosslinked dual-network hydrogels based on methacrylated gelatin (GelMA). *In vitro* characterization confirmed the hydrogel’s suitable morphological architecture, optical clarity, and excellent biocompatibility, establishing it as an optimal biological adhesive for sutureless graft implantation. This multi-lenticule encapsulation strategy using the hydrogels successfully reconstructed experimental rabbit corneal defects (7.0-mm diameter) *in vivo*. Over a 5-week postoperative period, the hydrogel demonstrated controlled biodegradation while maintaining structural integrity and optical functionality throughout tissue remodeling. It effectively adhered to the surrounding stromal tissues and supported epithelial regeneration over the transplanted grafts. The study demonstrates sutureless-free corneal stromal lenticule implantation, enabled by the GelMA-based photocrosslinked dual-network hydrogel, addressed the limitations of individual SMILE lenticules. The GelMA-based photocrosslinked dual-network hydrogel serves as both a biocompatible adhesive for multi-lenticule implantation and an optimal functional material for reconstructing corneal defects.

## 1 Introduction

Corneal blindness, defined as visual impairment caused by corneal damage, leading to scarring and vision loss, remains to be a major global public health concern, particularly prevalent in developing countries (Tidke and Tidake, 2022; Kate and Basu, 2023). Its etiological factors include traumatic injuries, infections, genetic disorders, metabolic abnormalities, developmental defects, and idiopathic causes (Tidke and Tidake, 2022). As the primary therapeutic intervention, corneal transplantation replaces damaged corneal tissue with donor grafts to restore vision (Kate and Basu, 2023; Tjoa et al., 2024). However, this procedure faces three principal limitations: severe donor shortages, a scarcity of trained surgeons, and the urgent demand for alternative solutions (Kate and Basu, 2023).

The corneal stromal lenticules extracted from the SMILE procedure serve as extracellular matrix (ECM) scaffolds, abundant in collagen, which are usually safe, effective, and cost-efficient, with high mechanical strength and transparency, becoming ideal candidates for corneal stroma replacement (Santra et al., 2022; Zhang et al., 2023). Clinical applications have demonstrated the efficacy of ECM scaffolds in treating various corneal pathologies including corneal ulcers, stromal defects, keratoconus, and perforations (Riau et al., 2020; Angunawela et al., 2012; Liu et al., 2015; Aghamollaei et al., 2024; Min Klimesova et al., 2024). As the lenticule’s thickness (<140 μm) and diameter (typically 6.6 mm) for ECM scaffolds cannot meet the transplantation requirements, stacking multiple lenticules becomes necessary to get adequate thickness and dimensions (Wu et al., 2015). However, conventional suture fixation may induce lenticule distortion during surgical manipulation, potentially causing postoperative astigmatism (Pagano et al., 2022). Therefore, sutureless corneal stromal lenticules transplantation based on tissue adhesion has become as an innovative advance in ophthalmic surgical applications.

Hydrogel biomaterials, as three-dimensional polymeric networks with ECM mimetic properties, offer structural and functional advantages for corneal regeneration (Catoira et al., 2019; Chameettachal et al., 2023). GelMA hydrogels selected for this study were specifically engineered for the corneal biocompatibility, water-soluble, non-immunogenic profile, predictable biodegradation, and interconnected porous architecture–critical parameters aligning with corneal tissue engineering requirements (Khosravimelal et al., 2021). Notably, the microporous framework enables precise cellular bioactivity modulation through enhanced cell-matrix interactions and dynamic nutrient-waste exchange via pore-derived channels (Cao et al., 2017; Oh et al., 2007). Furthermore, UV-responsive phototunability was achieved through the photoinitiator integration, generating double-network architecture while enhancing mechanical integrity beyond conventional single-network constructs (Aldana et al., 2019; Kilic Bekta et al., 2018; Li et al., 2018; Ichanti et al., 2020).

While previous studies have demonstrated the applications of GelMA-based hydrogels for sutureless corneal transplantation. Xuan Zhao’s research establishing their adhesive functionality in rabbit lamellar keratoplasty (Zhao X. et al., 2022) and Fuyan Wang’s exploration of stramal defect repair (Wang et al., 2023), however, critical gaps remain in their application spectrum. Crucially, the implementation of GelMA-based hydrogels in sutureless stromal lenticule transplantation remains unexplored territory. Our innovation centers on the photo-crosslinkable dual-network system that enables sutureless integration between hydrogels and stromal lenticules. In this paper, two corneal stromal lenticules were successfully implanted into the recipient corneal stromal bed. The remaining interfacial spaces were filled with hydrogels, which served dual functions as both a biocompatible filler and a biological adhesive. To the best of our knowledge, no previous work has ever studied the hydrogel applications for sutureless corneal stromal lenticules transplantation.

Therefore, in this paper, we aim to study this innovative approach facilitated seamless integration of the GelMA-based hydrogels, transplanted lenticules, and host stromal tissue into a unified composite structure through *in situ* photopolymerization. The innovative strategy could significantly advance multilayer corneal stromal lenticules transplantation techniques, ultimately leading to the clinical application for addressing the challenges posed by the scarcity of corneal donors.

## 2 Results and discussion

### 2.1 Synthesis of GelMA/HA-NB/LAP composite hydrogels

In this study, a photocrosslinked double-network hydrogel based on gelatin methacryloyl (GelMA) and nitrobenzyl-functionalized hyaluronic acid (HA-NB; hyaluronic acid functionalized with N-(2-aminoethyl)-4-(4-(hydroxymethyl)-2-methoxy-5-nitrosophenoxy) butanamide) was developed through rational biomaterial design. This approach aimed to address the limitations of conventional single-network hydrogels, such as insufficient interfacial integration in the moist ocular surface environment and weak resistance to dynamic mechanical stress (Hu et al., 2025). Inspired by the natural composition of the corneal ECM, which is primarily composed of type I collagen and glycosaminoglycans (Espana and Birk, 2020), the hydrogel was formulated with a specific ratio of GelMA to HA-NB (7% GelMA, 1.75% HA-NB) to mimic the native tissue architecture, thereby enhancing its biointegration capability with the host tissue (Wang et al., 2023).

**FIGURE 1:**
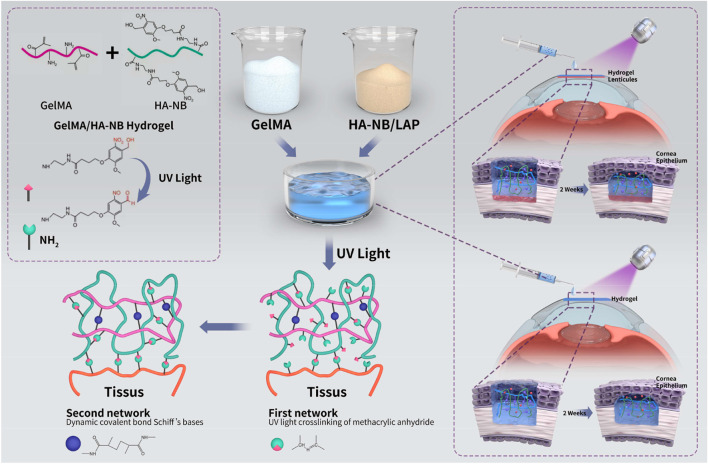
Schematic illustration of the chemical cross-linking mechanism of GelMA/HA-NB hydrogel and its application as an implant for the repair of corneal stromal defects.

Under the condition of 0.14% concentration of photoinitiator LAP (lithium phenyl-2,4,6-trimethylbenzoylphosphinate), exposure to ultraviolet light (365 nm) can induce the formation of an interpenetrating double-network (IDN) hydrogel between GelMA and HA-NB. As shown in Figure 1, the construction of the double network follows a two-step mechanism of “photolysis-covalent crosslinking”: the first network is formed by the preliminary self-assembly of GelMA and HA-NB through physical crosslinking or mild chemical crosslinking to create a precursor network; ultraviolet light triggers the photoinduced cleavage of NB groups (e.g., O-nitrobenzyl ether) in HA-NB, releasing highly reactive aldehyde groups (-CHO); the released aldehyde groups undergo a Schiff base reaction with residual amino groups (-NH_2_) in GelMA or exposed primary/secondary amines after hydrolysis, forming further crosslinks via C=N covalent bonds, resulting in a more mechanically stable double-network structure. The NB group acts as a photosensitive “molecular switch,” with its core functions being: before irradiation, the NB group exists as an “inert” linking moiety within the HA molecular chains, maintaining the initial material morphology; upon ultraviolet excitation, chemical bonds (e.g., C-O, C-N bonds) in the NB group undergo cleavage, releasing active functional groups such as aldehyde and carboxyl groups, which provide “chemical handles” for subsequent covalent crosslinking with the second network. (Wang et al., 2023; Dhand et al., 2021). The resulting hydrogel exhibits enhanced adhesiveness, controllable swelling behavior, and favorable biocompatibility (Wang et al., 2023; Dhand et al., 2021; Hong et al., 2019), offering a feasible strategy for suture-free closure in corneal surgery.

The core advantage of this design stems from the functional complementarity and synergy between GelMA and HA-NB at the molecular and cellular levels. The GelMA network not only provides a physical scaffold, but also effectively promotes the adhesion, proliferation, and migration of corneal stromal cells and epithelial cells through its retained native gelatin RGD (arginine, glycine, and aspartate) sequences and matrix metalloproteinase (MMP)-degradable motifs (Su et al., 2022). At the molecular level, RGD motifs specifically bind to cell-surface integrin receptors (e.g., α5β1 and αvβ3), activating focal adhesion kinase (FAK) and subsequently initiating key signaling pathways such as the mitogen-activated protein kinase (MAPK) pathway (Hersel et al., 2003; Schwartz and Assoian, 2001; Huang et al., 2021). This “RGD–integrin–FAK” signaling axis drives cytoskeletal reorganization, enhances mechanical anchoring between cells and the material, and provides a dynamically remodelable microenvironment via MMP-sensitive degradation, thereby laying a critical biological foundation for the active repair of corneal defects (Su et al., 2022; Klotz et al., 2016).

HA was selected as the backbone macromolecule. Photoresponsivity NB was linked to the backbone of HA, with LAP forming the component HA-NB system (Yang et al., 2016). This system exerts multifaceted regulatory effects during corneal wound healing, enhancing tissue hydration, maintaining corneal optical transparency, and improving local immune tolerance, all of which are crucial prerequisites for achieving functional corneal regeneration. HA-NB inherits the inherent high hydrophilicity and water-retention capacity of HA. As a major glycosaminoglycan component of the corneal stroma, HA possesses abundant hydrophilic groups (e.g., hydroxyl and carboxyl groups) on its molecular chains, endowing it with excellent moisture absorption, water-holding capacity, and unique viscoelasticity (Iaconisi et al., 2023; Srinivasan et al., 2023). Under physiological conditions, HA maintains a relatively high viscosity on the ocular surface during the inter-blink period, enabling prolonged retention and protective effects. When subjected to shear forces generated by blinking, its solution exhibits shear-thinning behavior, with viscosity rapidly decreasing, thereby facilitating uniform spreading of HA across the ocular surface. This dynamic rheological property helps enhance tear film stability, strengthen adhesion to the corneal epithelium, and reduce friction-induced ocular surface damage. Furthermore, HA molecules can efficiently bind water molecules via hydrogen bonding, which not only increases tear film thickness and maintains tear film homeostasis but also significantly improves the wettability of the corneal surface. Studies have confirmed that HA can also accelerate wound healing by promoting the migration of corneal epithelial cells (Srinivasan et al., 2023).

Regarding the maintenance of optical transparency, the HA-NB system is expected to reduce corneal opacity by inhibiting fibrotic remodeling, regulating myofibroblast activation, and optimizing stromal hydration. Following corneal injury, the abnormal differentiation of corneal stromal cells into myofibroblasts driven by transforming growth factor-β1 (TGF-β1) signaling, along with the resultant disordered collagen deposition, constitutes a key pathological process leading to corneal scar formation and loss of transparency (Muppala et al., 2019). Targeting this mechanism, HA-based biomaterials have demonstrated clear anti-fibrotic potential. For instance, the heavy chain-hyaluronic acid/pentraxin 3 (HC-HA/PTX3) complex purified from amniotic membrane can, under transforming growth factor-β1 (TGF-β1) stimulation, suppress the cytoplasmic expression of α–smooth muscle actin (α-SMA) and the nuclear translocation of phosphorylated Mothers Against Decapentaplegic Homolog 2/3 (pSMAD2/3) in human corneal fibroblasts and myofibroblasts, while reprogramming the cells toward a keratocan-expressing corneal stromal cell phenotype (Zhu et al., 2020). Their study provides direct molecular evidence for the scar-inhibiting effect of HA-based matrices.

HA-NB is also expected to enhance corneal immune privilege by regulating the immune microenvironment. Leveraging the intrinsic immune-privileged properties of the cornea, HA derivatives can promote the polarization of macrophages toward the anti-inflammatory M2 phenotype, characterized by upregulation of arginase-1 (Arg-1) and interleukin-10 (IL-10), along with downregulation of interleukin-12 (IL-12) (He et al., 2014; He et al., 2013; Chrostek and Cylwik, 2025). Furthermore, HA can suppress T-cell proliferation and reduce the release of pro-inflammatory cytokines such as interferon-γ (IFN-γ) and tumor necrosis factor-α (TNF-α), thereby mitigating immune-mediated damage and the risk of rejection (He et al., 2014; Chrostek and Cylwik, 2025).

In summary, the hydrogel designed in this study utilizes a photo-triggered dual-crosslinking mechanism that theoretically enables the simultaneous enhancement of its mechanical properties and wet tissue adhesion strength. Functionally, this system integrates the biomimetic cell-adhesive capacity endowed by GelMA with the multiple roles contributed by HA-NB—including dynamic hydration, anti-fibrotic activity, and immunomodulation—thereby establishing a composite platform with the potential to achieve both physical closure of corneal defects and transparent, functional tissue regeneration. Consequently, this work provides an innovative strategy with translational promise for addressing the challenges of poor interfacial integration and insufficient long-term functional recovery in corneal repair.

### 2.2 Characterization of hydrogels

#### 2.2.1 Fourier transform infrared spectroscopy (FTIR) analysis

**FIGURE 2:**
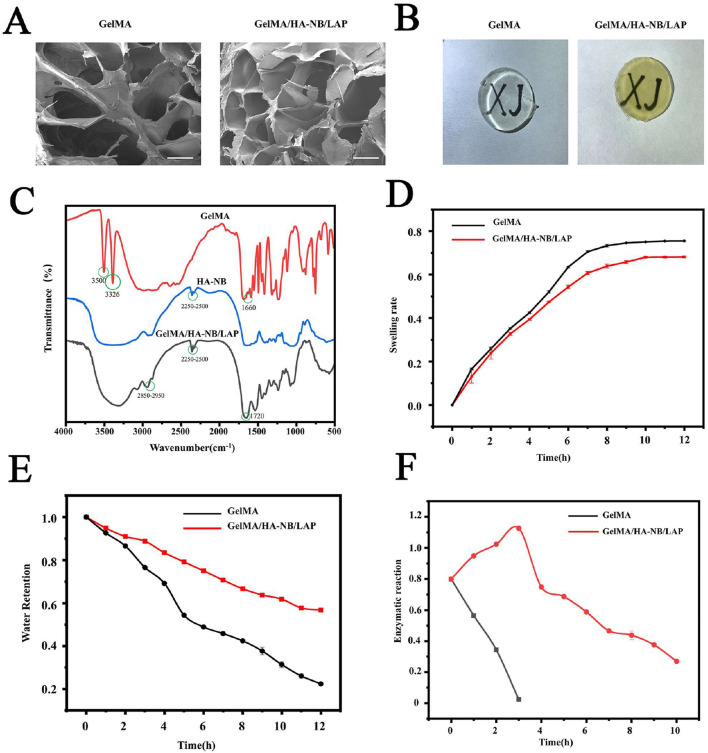
In vitro characterization and property determination of hydrogels. **(A)** Comparison of SEM images of hydrogels; **(B)** Observation of the overall macroscopic transparency of the hydrogel after UV irradiation; **(C)** FTIR image analysis; **(D)** Swelling rate of hydrogel in PBS at 37 °C; **(E)** Study of water retention properties of hydrogels at different time points under 37 °C; **(F)** Enzymatic reaction of hydrogel in collagenase solution (5 mg/mL) at 37 ℃. Scale bar: 100 μm.

The chemical structure and functional group evolution of the GelMA/HA-NB/LAP hydrogel were characterized in depth using FTIR, taking into account the structural features of its constituent polymers, GelMA and HA-NB. As shown in Figure 2C, a broad absorption peak in the range of 3326–3500 cm^-1^ appeared in the red curve, which is attributed to the stretching vibration of hydroxyl (-OH) groups in GelMA. In contrast, the absorption signal in the 2850–2950 cm^-1^ range was significantly enhanced in the black curve. This corresponds to the characteristic peak of the methylene (-CH_2_-) stretching vibration, indicating the close integration of polymer chains within the composite system. To elucidate the photo-responsive crosslinking mechanism, we focused on the spectral changes before and after photolysis.

Before photolysis (HA-NB, blue curve), a characteristic peak observed at ∼2250 cm^-1^ was closely associated with the “unphotolyzed state” of the NB group. Regarding the assignment of this peak, the carbon-carbon double bond (C=C) stretching vibration of standard alkenes typically appears in the 1620–1680 cm^-1^ range, while the 2250 cm^-1^ region is commonly associated with nitrile groups (C≡N). However, this peak does not correspond to a nitrile group in the present system. Given that the NB group possesses an O-nitrobenzyl ether (HA-O-CH_2_-Ph-NO_2_) structure, the characteristic peak at ∼2250 cm^-1^ is more likely attributed to the overtone vibration of the nitro group (-NO_2_) (whose fundamental frequencies are located at 1530 cm^-1^ and 1350 cm^-1^, with overtone peaks extending into the 2200–2300 cm^-1^ range), or a weak overtone response arising from the synergistic effect of the ether bond and the nitro group (where the main ether bond peak is at 1000–1300 cm^-1^, but weak overtone peaks can extend to ∼2200 cm^-1^).

After UV irradiation (GelMA/HA-NB/LAP, black curve), the characteristic peak at ∼2250 cm^-1^ disappeared completely, while a new characteristic peak emerged at 1720 cm^-1^. The peak at 1720 cm^-1^ is definitively assigned to the stretching vibration of the aldehyde carbonyl (C=O). This spectral “trade-off”—specifically, the disappearance of the peak at ∼2250 cm^-1^ and the appearance of the peak at 1720 cm^-1^—directly corroborates the photolysis process of the NB group: UV irradiation triggered the photocleavage of the NB group, resulting in the release of active aldehyde groups.

In summary, the NB group functions as a “photo-controlled chemical switch” within this system. The FTIR results clearly record the transformation process from ∼2250 cm^-1^ (characteristic signal of the NB group before photolysis) to 1720 cm^-1^ (signal of the aldehyde C=O after photolysis). This not only verifies the cross-scale regulation mechanism of “light signal → chemical bond → network structure” but also clarifies the internal crosslinking mechanism of the hydrogel: specifically, under UV irradiation, the aldehyde groups released from the NB group rapidly undergo a Schiff base reaction with the amino groups on GelMA and the tissue surface, generating stable imine bonds and thereby constructing a robust double-network hydrogel structure.

#### 2.2.2 Morphological analysis of hydrogels

The hydrogel synthesized in this study exhibited a porous structure, as evidenced by scanning electron microscopy (SEM) analysis (Figure 2A). Notably, the addition of HA-NB did not compromise the porous morphology, as the GelMA/HA-NB/LAP composite exhibited displayed a structure compared to that of pure GelMA. Furthermore, HA-NB and amine-containing components enabled the formation of a dual-network structure via UV light-mediated crosslinking (Hong et al., 2019). This hierarchically interconnected porosity not only fosters cell proliferation while enabling efficient transport of nutrients, metabolites, and oxygen but also ensures seamless nutrient diffusion from the host corneal tissue to encapsulated lenticules (Wu et al., 2024). The interconnected networks are crucial for the hydrogel’s performance in various applications, particularly in tissue engineering.

#### 2.2.3 Macroscopic transparency of hydrogels

Optical transparency of disc-shaped UV-crosslinked hydrogels was assessed through a visualization test, wherein samples were placed over the “XJ” lettering (test pattern) printed in black ink on white background (Figure 2B). Although the strategic integration of NB functional groups imparted a slight yellowish tint to the hydrogels, this optical benign coloration remained compatible with the transmittance specifications mandated for corneal applications. This qualitative observation is supported by quantitative data from prior studies, which report that such GelMA/HA-NB/LAP hydrogels exhibit a visible light transmittance of approximately 78% across 300–800 nm—a value that closely matches that of the native human cornea (Wang et al., 2023). Together, these results confirm that the prepared hydrogels possess the critical optical properties necessary for potential use in corneal repair.

#### 2.2.4 Swelling rate of hydrogels

Swelling rate emerge as crucial determinants in ocular tissue repair, particularly in minimizing astigmatism and maintaining corneal sphericity following corneal applications. In our studies, the swelling rates of GelMA and GelMA/HA-NB/LAP hydrogels were assessed. The findings indicated that both hydrogels achieved swelling equilibrium at approximately 8 h, with the GelMA/HA-NB/LAP hydrogel exhibiting a consistently lower swelling rate compared to the GelMA hydrogel (Figure 2D). Photocrosslinking-induced dual-network architecture enabled GelMA/HA-NB/LAP to achieve a more compact microstructure, which absorbed less water, resulting in a reduced swelling rate (Hong et al., 2019; Hwang et al., 2015). For corneal stromal lenticules transplantation, the lower swelling rate is beneficial for reducing sugically induced astigmatism.

#### 2.2.5 Water retention of hydrogels

As evidenced by quantitative analysis (Figure 2E), the GelMA/HA-NB/LAP composite demonstrated superior water retention capacity than GelMA alone. The critical role of ocular surface moisture in maintaining eye health, preventing ocular diseases, and treating surface-related conditions is paramount. Owing to the porous structure, GelMA/HA-NB/LAP exhibited enhanced water retention capabilities, effectively meeting the requirements for water absorption and wound protection.

#### 2.2.6 Enzymatic degradation of hydrogels

Biodegradability represents a critical consideration in scaffold-based corneal stromal engineerin. An ideal cell carrier should degrade progressively alongside the extracellular matrix secretion by the delivered cells, enabling the nascent matrix to gradually replace the biomaterial and ultimately form a tissue that closely resembles the native structure and function. During this process, the interplay between the mechanical properties of the scaffold and its degradation behavior is particularly crucial and requires precise modulation to facilitate proper healing of the injured tissue. Furthermore, the degradation characteristics of a scaffold are closely linked to its swelling behavior and water content, parameters that directly influence the material’s biocompatibility and cellular growth (Orash Mahmoudsalehi et al., 2025).

Based on this rationale, the enzymatic degradation of the GelMA/HA-NB/LAP composite hydrogel was analyzed and compared with pure GelMA. As shown in Figure 2F, in the presence of collagenase (5 mg/mL in PBS, pH 7.4, 37 °C), the GelMA/HA-NB/LAP composite demonstrated significantly enhanced degradation resistance, with an enzymatic resistance time extending to approximately 10 h, whereas pure GelMA maintained integrity for only 3 h. This slow-degrading characteristic helps preserve the necessary mechanical support during the period when cells secrete and assemble new extracellular matrix, reducing the risk of premature loss of structural integrity and thereby promoting orderly tissue remodeling and the formation of a transparent matrix.

Animal studies corroborated these findings, demonstrating that GelMA/HA-NB/LAP retained structural integrity in the ocular environment for a minimum of 5 weeks without lenticule loss. Comprehensive data from these *in vivo* evaluations will be comprehensively detailed in forthcoming sections. It should be noted that the degradation of collagen and other extracellular matrix components in the intraocular environment is primarily mediated by the matrix metalloproteinase (MMP) family (e.g., MMP-1, MMP-2, MMP-9) and strictly regulated by their tissue inhibitors (TIMPs) (Sivak and Fini, 2002; Jia et al., 2014; Garg et al., 2025; Folorunso et al., 2025). Even under pathological conditions such as diabetic retinopathy, where MMP expression may be upregulated, their concentrations in ocular fluids remain typically in the ng/mL range—for instance, the average concentration of MMP-9 in the ocular fluid of diabetic patients is only 0.67 ± 0.66 ng/mL (Ünal et al., 2022). This is approximately 10^6^-fold lower than the mg/mL-level collagenase concentration used in this study. Therefore, despite the stringent conditions of the *in vitro* degradation assay, the significantly prolonged resistance to degradation demonstrated by the composite hydrogel in the *in vivo* environment strongly underscores that its degradation kinetics are better aligned with the timeline of tissue regeneration. This controlled retention stability is paramount to ensuring its successful support for cell-mediated scaffold remodeling and the ultimate achievement of functional corneal stromal regeneration.

The GelMA/HA-NB/LAP composite hydrogel developed in this study, owing to its controllable slow-degradation properties, achieves close alignment between scaffold retention duration and tissue regeneration progression, offering an effective material-design strategy for corneal stromal engineering. In contrast, conventional biological scaffolds such as human amniotic membrane (HAM) often degrade too rapidly before complete corneal healing, and their short-term degradation behavior fails to provide stable mechanical support throughout the repair process (Boroumand et al., 2024).

### 2.3 *In vitro* cell experiment

#### 2.3.1 Morphological and immunofluorescence identification of cells

**FIGURE 3:**
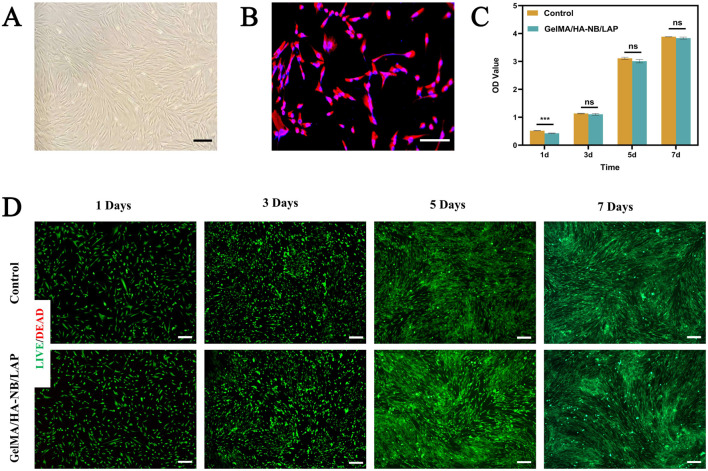
Identification of HCFs and in vitro cytocompatibility assessment of GelMA/HA-NB/LAP. **(A)** Morphological identification of HCFs; **(B)** Immunofluorescence identification of HCFs; **(C)** CCK- 8 assay of HCFs cultured in complete medium (control group) and extracts of GelMA/HA-NB/LAP (experimental group) for 1, 3, 5 and 7 days (*** p <0.001, n ≥ 3); **(D)** Live/dead staining assay of HCFs cultured in complete medium (control) and extracts of GelMA/HA-NB/LAP (experimental group) for 1, 3, 5 and 7 days. Scale bar: 100 μm.

On day 7 of primary culture, cells isolated from human corneal stromal lenticules exhibited elongated spindle-shaped or irregular triangular morphology under inverted microscopy (Figure 3A). Following subculture to passage 2 (P2), immunofluorescence staining revealed distinct red fluorescence within the cytoplasm (Figure 3B) with positive vimentin immunoreactivity. These findings collectively confirm the identity of the cells as human corneal fibroblasts.

#### 2.3.2 Cytocompatibility of GelMA/HA-NB/LAP

Our findings demonstrate that, given the enhanced physical characteristics of GelMA/HA-NB/LAP hydrogels relative to GelMA hydrogels, a systematic evaluation of their cellular interactions is imperative. To assess biocompatibility, HCFs were continuously cultured with GelMA/HA-NB/LAP hydrogel extracts (experimental group) or standard complete medium (control group), followed by CCK-8 assays and live/dead staining. The CCK-8 results (Figure 3C) revealed a mild suppression of cell proliferation in the experimental group on day 1, with a statistically significant reduction in cell viability (p < 0.001). However, viability levels recovered and were comparable to the control on days 3, 5, and 7 (p > 0.05), confirming the hydrogel’s good cytocompatibility.

LAP, a visible-light photoinitiator, generally exhibits good water solubility and cytocompatibility (Xu et al., 2020). However, studies have also indicated that unreacted methacrylated monomers and residual photoinitiators in photocrosslinked systems may introduce cytotoxic risks (Nguyen et al., 2019). In the present study, the hydrogel extract group showed a decrease in cell activity on day 1, followed by gradual recovery thereafter. This trend aligns with reports in the literature, wherein aqueous extracts of photopolymerized materials can release water-soluble toxic small molecules initially, and their toxicity can be significantly reduced after immersion in an aqueous medium (Ovsianikov et al., 2011). Therefore, we propose that the early decline in cell activity mainly results from the concentrated release of residual photoinitiator or un-crosslinked monomers during the initial extraction phase, rather than from long-term toxic effects caused by degradation products of the hydrogel matrix itself. Prior to clinical translation, extending dialysis or implementing other post-processing steps can effectively remove soluble residual components, thereby reducing such initial toxicity risks.

Complementary live/dead staining assays (Figure 3D) provided spatially resolved assessment of cellular vitality. Fluorescence imaging confirmed consistently high viability levels across all timepoints, demonstrating the material’s biocompatibility.

Collectively, these findings reveal a characteristic adaptation phase where initial cell-material interactions trigger reversible metabolic adjustments without compromising long-term survival. The complete normalization of viability parameters within 72 h, coupled with absence of cumulative cytotoxicity, positions GelMA/HA-NB/LAP as a structurally and biologically optimized substrate for corneal stromal lenticule transplantation. This biphasic biocompatibility patter—transient acclimatization followed by robust cytocompatibility—suggests particular utility in ocular regenerative therapies.

### 2.4 Rabbit *in vivo* model of corneal stromal defect

#### 2.4.1 Evaluation of GelMA/HA-NB/LAP in lenticules transplantation in rabbits

To validate the translational potential of GelMA/HA-NB/LAP in corneal stromal lenticule transplantation, we established a rabbit corneal stroma defect model (7.0 mm diameter, 200 μm depth). In the experimental group, two SMILE-derived lenticules were implanted into the recipient stromal bed. Residual interface gaps were filled with GelMA/HA-NB/LAP hydrogel and subsequently photo-crosslinked *in situ* using UV light (365 nm, 12 W) for 5 min. This irradiation time was consistent with that reported in prior studies which employed higher-power UV light (365 nm, 250 W) and showed no significant adverse ocular effects in rabbits (Li et al., 2023). The substantially lower power (12 W) used here at the same duration ensures comparable crosslinking while further minimizing any potential phototoxic risk, thereby enhancing the procedural safety profile.

An ideal corneal adhesive must meet the following key requirements: (1) superior wet adhesion; (2) controllable gelation time; (3) good biocompatibility with no induction of corneal vascularization; (4) high transparency; and (5) capacity for *in vivo* degradation (Rosenquist et al., 2023).

**FIGURE 4:**
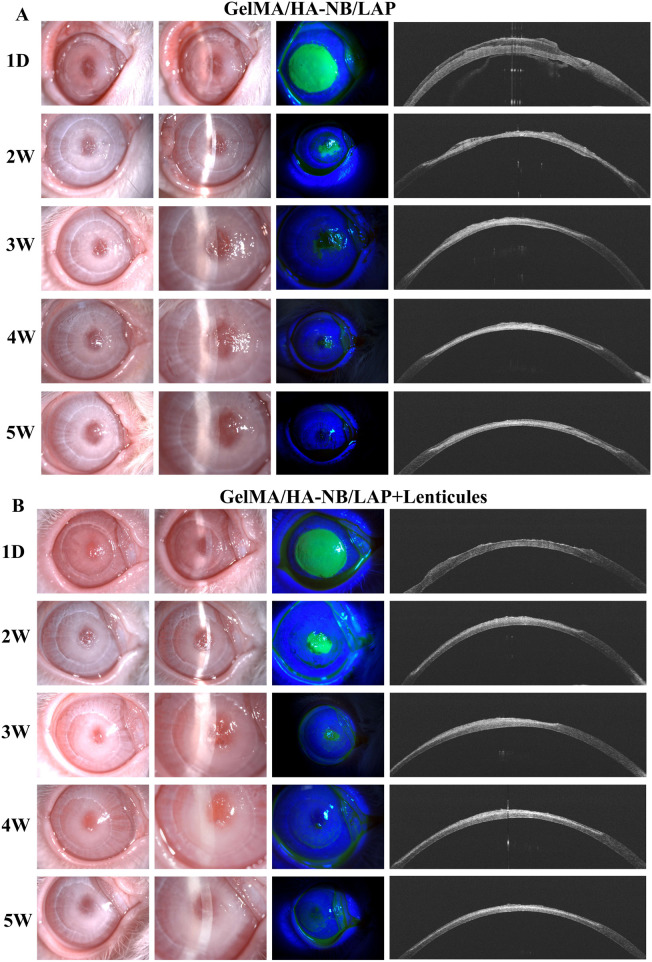
Postoperative evaluation of corneal stromal defect repair using hydrogel alone or in combination with implanted lenticules. **(A)** Hydrogel group: Rabbit corneas were assessed in series at postoperative day 1 and weeks 2, 3, 4, and 5 (from left to right) via slit-lamp examination (diffuse and slit illumination), sodium fluorescein staining, and anterior segment optical coherence tomography (OCT). **(B)** Hydrogel‑lenticules combination therapy group: The evaluation time points and methodologies were identical to those described for group **(A)**.

Adhesive strength serves as a critical performance metric for bio-tissue adhesives. As shown by the slit lamp results (Figure 4), throughout the experiment, the hydrogel effectively filled and tightly adhered to a 7 mm corneal stromal defect, with no observed displacement or detachment of the donor corneal stromal lenticule, demonstrating its excellent wet adhesion. The high adhesive strength of this hydrogel arises from its internally formed double-network crosslinked structure. This property addresses a key limitation of clinically commonly used fibrin glue—due to insufficient adhesive force, fibrin glue is only suitable for sealing corneal perforations ≤3 mm in diameter and cannot serve as a replacement for traditional surgical sutures (Sharma et al., 2023; Deshmukh et al., 2020).

The tunability of gelation time represents another critical factor. The GelMA/HA-NB/LAP hydrogel exhibits a suitable gelation duration, providing surgeons with ample time for precise adjustment of graft positioning. This advantage effectively addresses the limitations in operational time control associated with cyanoacrylate adhesives and fibrin glue (Sharma et al., 2023; Deshmukh et al., 2020).

Analysis of diffuse illumination and narrow-slit lamp images revealed favorable clinical outcomes in both hydrogel-treated groups (Figure 4), with no significant corneal edema, immune rejection, or neovascularization observed in the rabbit eyes post-operation. This outcome can be attributed to the excellent biocompatibility of the hydrogel, a property further confirmed by prior *in vitro* cell experiments. This advantage effectively circumvents the risks of potential inflammatory responses and tissue necrosis associated with the intrinsic toxicity of cyanoacrylate-based corneal adhesives (Sharma et al., 2023). Under slit-lamp examination, the GelMA/HA-NB/LAP hydrogel appeared pale yellowish and nearly colorless, indicating its desirable optical transparency. Corneal transparency primarily depends on the regular arrangement of collagen fibrils and the compositional ratio between collagen and proteoglycans within the ECM (Meek and Knupp, 2015). The GelMA/HA-NB/LAP hydrogel developed in this study exhibits a high degree of biomimicry to the corneal ECM in its composition, being primarily constituted of gelatin and hyaluronic acid. Gelatin, a hydrolyzed derivative of collagen, and hyaluronic acid, a key polysaccharide component of the ECM, collaboratively create a biomimetic microenvironment that mirrors the structural characteristics of the native corneal ECM (Meek and Knupp, 2015; Kesharwani et al., 2024). This provides a favorable foundation for corneal tissue repair.

As shown in Figure 4, fluorescein sodium staining demonstrated a gradual decrease in green fluorescence at the center of the corneal defect over time, indicating continuous regeneration of corneal epithelial cells, with complete epithelial healing achieved by the fifth week post-operation. Further evaluation via anterior segment optical coherence tomography (OCT) confirmed the progressive biodegradation of the implanted hydrogel *in vivo*, thereby addressing the non-degradable limitation inherent to cyanoacrylate-based adhesives. Moreover, in the group receiving the combined GelMA/HA-NB/LAP hydrogel and stromal lenticule implantation, OCT imaging revealed smoother and more regular corneal contour curves compared with the hydrogel-only group. This improvement can be attributed to the integrated lenticule, which, owing to its excellent mechanical properties, provides enhanced structural support to the cornea, thereby optimizing its overall biomechanical characteristics (Santra et al., 2022).

**FIGURE 5:**
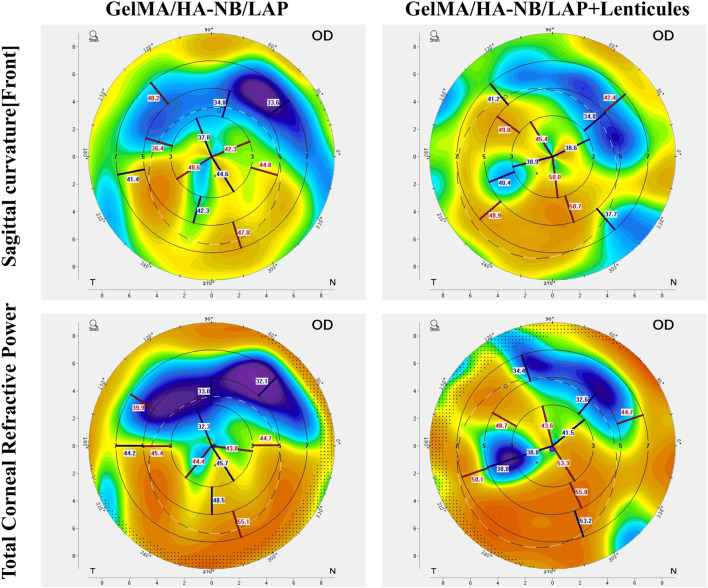
Comparative analysis of corneal topography at postoperative week 5. Topographic maps of rabbit corneas following treatment of stromal defects with hydrogel alone or in combination with transplanted lenticules.

Traditional suturing techniques are prone to inducing irregular astigmatism, a type of astigmatism that cannot be corrected by conventional spherical or cylindrical lens, often resulting in reduced postoperative visual quality (Pagano et al., 2022; Busin et al., 1998; Ueno et al., 2021). To evaluate the effect of the composite hydrogel on corneal morphology following the repair of corneal stromal defects, a qualitative analysis of postoperative corneal topographic changes was conducted. The results showed that corneas sealed with the adhesive exhibited a largely symmetrical and regular astigmatism pattern. Further observation revealed that this combination therapy resulted in superior corneal surface symmetry during wound remodeling compared to the hydrogel-only treatment group (Figure 5), suggesting synergistic benefits from the lenticule-hydration matrix interaction in maintaining corneal topography.

The primary limitation of this study lies in the insufficient observation period. Corneal healing is a prolonged and dynamic process, with peaks in opacification and stromal remodeling often occurring during the subacute or later stages. The current 5-week observation mainly evaluated the early biocompatibility and initial healing outcomes of hydrogel-assisted adhesion. Although the results are positive, they are not sufficient to comprehensively assess the long-term efficacy of this technique. The key to successful corneal transplantation is long-term optical stability, as late-stage fibrosis or hydrogel degradation may affect transparency and refractive status. For instance, Zheng et al. reported the occurrence of degradation-related opacities in rabbit corneas 6 months after implantation of poly (ethylene glycol)/poly (acrylic acid) (PEG/PAA) hydrogels (Zheng et al., 2015). Therefore, follow-up studies must extend the follow-up duration, with a focus on evaluating changes in corneal light transmittance, to confirm the long-term safety and effectiveness of this adhesive system.

#### 2.4.2 Histopathological evaluation (H&E) of corneal tissue

**FIGURE 6:**
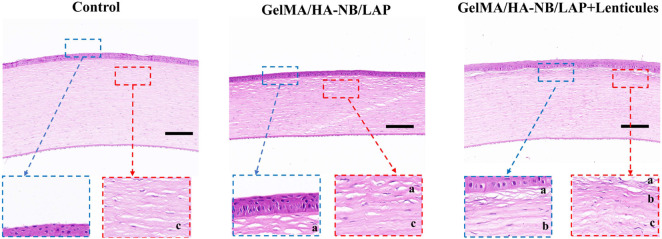
Hematoxylin and eosin (H&E) staining of corneal tissues at postoperative week 5. (a) GelMA/HA‑NB/LAP hydrogel region, (b) transplanted lenticules region, (c) host rabbit corneal tissue. Scale bars: (top: 200 μm, bottom: 100 μm).

Histopathological evaluation of corneal repair efficacy was performed at 5 weeks postoperatively through HE staining, comparing therapeutic outcomes between hydrogel-only treatment and combination therapy with hydrogel-corneal lenticule constructs. As shown in Figure 6, both groups demonstrated successful graft-host integration characterized by seamless tissue continuity without discernible interfacial gaps. Notably, complete epithelialization was observed across all repaired corneal defects, with newly formed epithelial layers exhibiting normal morphological characteristics. These findings collectively substantiate the histocompatibility of the GelMA/HA-NB/LAP bioadhesive, while the preserved structural integrity and physiological remodeling processes observed in both groups provided evidences for its clinical potential in ocular surface reconstruction.

#### 2.4.3 SEM assessment of corneal tissue

**FIGURE 7:**
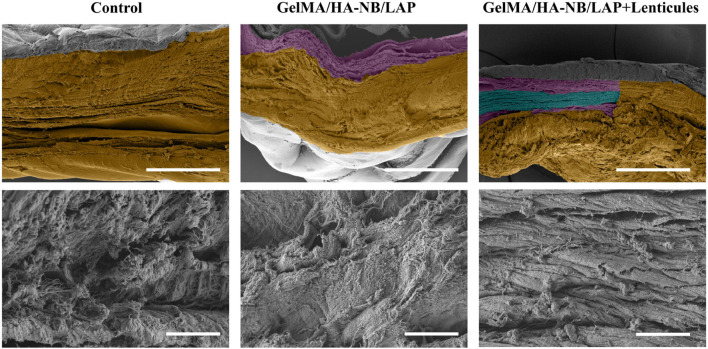
Scanning electron microscopy (SEM) observation of rabbit corneal tissues at postoperative week 5. Yellow: host rabbit corneal tissue; purple: GelMA/HA‑NB/LAP hydrogel; green: transplanted lenticules. Scale bars: (top: 200 μm, bottom: 10 μm).

At 5 weeks postoperative, scanning electron microscopy (SEM) analysis demonstrated robust integration of the GelMA/HA-NB/LAP hydrogels and corneal stromal lenticules within the injury site, with complete filling of the corneal defect observed (Figure 7). The graft-host interface exhibited seamless structural continuity, characterized by tight adhesion between the implanted materials and recipient stromal bed without detectable gaps or delamination. These findings suggsted the double-network architecture of the GelMA/HA-NB/LAP hydrogels enhanced mechanical stability while promoting bioadhesion to stromal bed. Notably, the observed tissue-hydrogel-lenticule integration aligns with the material’s capacity to mimic native stromal extracellular matrix properties, facilitating cellular infiltration and remodeling processes. This bioengineered strategy not only advances corneal stromal regeneration but also establishes a translational framework for optimizing lenticule transplantation outcomes in patients with corneal defects. The results collectively validate GelMA/HA-NB/LAP hydrogels combined with corneal stromal lenticules as a clinically promising bioadhesive platform for enhancing corneal repair through dual mechanisms of structural reinforcement and biomimetic tissue integration.

## 3 Conclusion

This study presents a photocrosslinked double-network GelMA/HA-NB/LAP hydrogel as an innovative bioadhesive for sutureless corneal stromal lenticule transplantation. The hydrogel was synthesized through a streamlined fabrication protocol and demonstrated well-defined physicochemical properties. *In vitro* assessments confirmed the biocompatibility, and robust support for human corneal fibroblasts proliferation. *In vivo* validation further highlighted the hydrogel’s multifunctional utility: it enabled secure, suture-free adhesion of multiple lenticules, achieved contouring of stromal irregularities, and facilitated corneal re-epithelialization.

Notably, the synergistic integration of GelMA/HA-NB/LAP with lenticules not only restored corneal transparency but also mimicked native stromal biomechanics, creating a permissive microenvironment for host tissue remodeling. These findings position the GelMA/HA-NB/LAP + lenticules as a transformative strategy for corneal regeneration, combining structural reinforcement, surgical simplification, and biological activity to address critical challenges in ophthalmic tissue engineering.

## 4 Materials and methods

### 4.1 Materials

GelMA (100–2000 kDa) and hyaluronic acid (HA) were obtained from Shanghai Aladdin Biological Reagent Co., Ltd. (China). MD44-3500 dialysis bags came from JielePu (United States). Supplementary materials comprised lithium phenyl-2,4,6-trimethylbenzoylphosphinate (LAP, Macklin, China), collagenase type I (Solarbio, China), and 1-Hydroxybenzotriazole (HOBt) supplied by Hubei Jiufenglong Chemical Co., Ltd. (China). N-(2-aminoethyl)-4-(4-(hydroxymethyl)-2-methoxy-5-nitrosophenoxy)-butanamide (NB) was procured from Shanghai Nafu Biotech (China). Cell culture reagents included DMEM-F12 medium (Gibco, United States) and fetal bovine serum (Vivacell, China). Biological detection agents featured anti-vimentin rabbit pAb, goat anti-rabbit IgG, CCK-8 assay kit, and live/dead cell double staining kit (all from Proteintech, China). Additional supplies contained 1-(3-dimethylaminopropyl)-3-ethylcarbodiimide (EDC, Macklin) and hematoxylin-eosin staining kit (H&E, Proteintech). Remaining chemicals were either domestically produced or internationally sourced.

### 4.2 Preparation of hydrogels

#### 4.2.1 Synthesis of HA-NB

The HA-NB conjugate was prepared through the following procedure: HA (408 mg, 1 mmol), NB (224 mg, 0.69 mmol), and HOBt (153 mg, 1 mmol) were mixed in 50 mL of distilled water under ambient conditions (Zhou F. et al., 2020; Luo et al., 2023; Liao et al., 2020). After adjusting the solution pH to 4.5, EDC (200 mg, 1.04 mmol) was gradually added, followed by continuous stirring for 48 h at room temperature. The reaction mixture was subsequently subjected to dialysis using membranes immersed in 0.1 M NaCl-HCl buffer (pH 3.5) for 48 h, then transferred to pure deionized water for another 48-h dialysis. The purified product was freeze-dried to obtain HA-NB as a powdered material.

#### 4.2.2 Precursor preparation of the hydrogels

To prepare the GelMA/HA-NB/LAP hydrogel precursors, lyophilized GelMA and HA-NB powders were first solubilized in phosphate-buffered saline (PBS) at 40 °C under continuous stirring. The photoinitiator LAP was subsequently supplemented into the homogeneous mixture. The optimized formulation contained 7% (w/v) GelMA blended with 1.75% (w/v) HA-NB and 0.14% (w/v) LAP, which was then aliquoted into light-protected vials and refrigerated at 4 °C for subsequent applications (Wang et al., 2023; Zhou F. et al., 2020). Photocrosslinking was achieved through UV exposure (365 nm wavelength, 12 W intensity) for 5 min to form stable hydrogel networks.

#### 4.2.3 FTIR analysis

Fourier-transform infrared spectroscopy serves as a robust analytical method for identifying molecular vibrations and functional group characteristics. The chemical compositions of GelMA, HA-NB, and their composite hydrogel were examined using an FTIR spectrometer (Nicolet iS20, Thermo Scientific, United States). Spectral data was collected across the 400–4000 cm^-1^ wavelength range to characterize molecular interactions and structural features.

#### 4.2.4 SEM of hydrogels

The lyophilized GelMA and GelMA/HA-NB/LAP specimens were secured on a conductive holder and subjected to 120-s gold sputter coating using a precision ion coater (DII-29030SCTR Smart Coater, JEOL, Japan). Subsequent microstructural evaluation was performed through field-emission scanning electron microscopy (SEM, JSM-7610FPlus, JEOL, Japan) to characterize surface topography and architectural features.

#### 4.2.5 Macroscopic transparency evaluation

Circular hydrogel specimens were positioned above a black 'XJ’ symbol printed on white background paper for visual documentation and transparency assessment according to established methodology (Wang et al., 2023).

#### 4.2.6 Swelling ratio of the hydrogels

Pre-weighed hydrogel samples (0.1 g each of GelMA and GelMA/HA-NB/LAP) were submerged in PBS solution maintained at 37 °C until reaching maximum swelling capacity. After carefully blotting surface moisture, the saturated hydrogels were measured as wet mass (W_W)_. The swelling ratio was determined through gravimetric analysis using the initial dry weight (W_D_) in the equation:

$$\text{Swelling ratio }\left(\%\right)=\frac{W_{W}-W_{D}}{W_{D}}\times 100\%$$

#### 4.2.7 Water retention analysis

To assess water retention capacity, hydrogel specimens with accurately measured identical quantities were first brought to saturation equilibrium under ambient conditions. The stabilized mass was documented as W_0_ before transferring samples to a temperature-controlled chamber maintained at 37 °C. Periodic mass determinations (Wt) were conducted at designated intervals to quantify temporal variations, with moisture retention efficiency computed through the established formula:

$$\text{Water Retention }\left(\%\right)=\frac{W_{t}}{W_{0}}\times 100\%$$

#### 4.2.8 *In vitro* enzymatic degradation

For evaluating GelMA and GelMA/HA-NB/LAP hydrogel decomposition, specimens were initially weighed (W_D_) to 0.1 g and immersed in collagenase type I solution (5 mg/mL PBS) at physiological temperature. Following surface moisture removal at scheduled time points, residual mass (W_W)_ was recorded to determine biodegradation extent using the computation:

$$\text{Mass Loss Ratio }\left(\%\right)=\frac{W_{D}-W_{W}}{W_{D}}\times 100\%$$

### 4.3 *In vitro* cellular assays

#### 4.3.1 Acquisition of human corneal lenticules

The corneal lenticules employed in this study were surgically harvested during SMILE procedures (Zhao J. et al., 2022) performed at the First Affiliated Hospital of Xinjiang Medical University. All corrective surgeries utilized the VisuMax femtosecond laser system (Carl Zeiss Meditec, Jena, Germany), operating at a repetition rate of 500 kHz with 130 nJ pulse energy. Surgical parameters included corneal caps configured with cap thicknesses varying between 100 and 120 μm and diameters spanning 7.4–7.9 mm. The extracted lenticular tissues demonstrated diameters of 6.5–6.9 mm with thickness measurements of 100–140 μm (Zhou X. et al., 2020). A superiorly positioned 2-mm incision was created at 135° for surgical access. These vision correction operations addressed either myopia or myopic astigmatism (Zhao J. et al., 2022), with all participants providing written informed consent prior to intervention. The research protocol received formal approval from the Ethics Committee of the First Affiliated Hospital of Xinjiang Medical University (Approval No. 231124-03), ensuring strict compliance with established ethical guidelines throughout the investigation in the Declaration of Helsinki.

#### 4.3.2 Isolation and cultivation of HCFs

The lenticular tissues underwent washing with phosphate-buffered saline (PBS) containing 1% penicillin/streptomycin for 10 min. Following this, the specimens were finely dissected and placed into centrifuge tubes filled with collagenase type I solution (2.0 g/L concentration). Enzymatic digestion proceeded for 5 h under controlled conditions (37 °C, 5% CO_2_ atmosphere), continuing until 90% tissue dissociation was achieved. The cellular suspension underwent initial centrifugation (1000 revolutions per minute, 5-min duration) with subsequent supernatant removal. Pelleted cells were reconstituted in 3 mL PBS solution and subjected to secondary centrifugation under identical parameters. Following complete supernatant aspiration, isolated HCFs were maintained in DMEM/F-12 growth medium enriched with 10% fetal bovine serum and 1% antibiotic solution. Cellular cultures were preserved in a humidified 5% CO_2_ incubator at 37 °C, with medium replacement occurring at 48–72 h intervals. Subculturing at 1:3 ratios was performed upon reaching 80% confluency (Bai et al., 2018). Experimental procedures utilized HCFs between passage 2 and 5 to ensure cellular consistency.

#### 4.3.3 Characterization of HCFs morphology

HCFs were monitored on a daily basis using an inverted phase-contrast microscope (model IX71-12FL/PH, Olympus, Japan). Key cellular characteristics including attachment patterns, structural features, and proliferative activity were systematically documented.

#### 4.3.4 HCFs immunofluorescence assay

HCFs were initially cultured in 3.5 cm dishes. After cellular attachment, specimens underwent three sequential 30-s rinses using phosphate-buffered saline (1×PBS), then fixed by immersing in 4% paraformaldehyde (PFA) for 30 min at ambient temperature (Yuan et al., 2025). Three additional PBS washes lasting 5 min each were subsequently administered. Membrane permeabilization involved 20-min exposure to 2% Triton X-100 solution, succeeded by triple 5-min PBS rinses. A 5% bovine serum albumin (BSA) blocking buffer was applied for 60 min at room temperature. Primary antibody solution (Anti-Vimentin Rabbit pAb, 1:100 dilution in blocking buffer) was introduced for overnight incubation at 4 °C. Following three 5-min PBS washes, secondary antibody (Goat anti-Rabbit IgG) treatment occurred under light-protected conditions for 60 min, with subsequent triple PBS rinses. 4,6-diamidino-2-phenylindole (DAPI) staining was performed for nuclear visualization. Samples were observed under an inverted fluorescence microscope (Leica DMI8, Germany) and images were captured at ×100 magnification. Vimentin signals appeared as red fluorescence, while nuclei were counterstained with DAPI (blue). The red and blue fluorescence images were merged using ImageJ 1.8 software (United States).

#### 4.3.5 *In vitro* cytotoxicity assessment

The coverslip application protocol involved precise placement to minimize sample disturbance while maintaining optical clarity. Post-staining procedures included thorough rinsing steps before transferring specimens to the microscope stage. Imaging parameters were optimized using the manufacturer’s software to capture detailed cellular morphology, with particular attention given to preserving fluorescence signal integrity during observation.

The proliferative effects of GelMA/HA-NB/LAP on HCFs were analyzed through CCK-8 testing. Lyophilized hydrogel samples underwent steam sterilization before being soaked in DMEM/F-12 containing 10% fetal bovine serum and 1% penicillin-streptomycin solution for 7 days to prepare extraction media. Cells were plated in 96-well culture dishes at 2,000 cells/well and allowed to adhere during 24-h incubation under standard culture conditions (37 °C, 5% CO_2_) (Saadh et al., 2025). The culture medium was then replaced with 200 µL of hydrogel extract, which was renewed every 72 h. At designated time points (days 1, 3, 5, 7), the extraction medium was removed and replaced with fresh medium containing 10% CCK-8 reagent. Optical density measurements at 450 nm were recorded using a Thermo MultiskanGO microplate reader. Parallel cultures maintained in complete medium without hydrogel extracts served as experimental controls (Wang et al., 2023).

#### 4.3.6 Assessment of *in vitro* cellular viability

The survival capability of HCFs when exposed to GelMA/HA-NB/LAP composites was assessed through dual fluorescence viability testing. Fibroblasts were plated in 96-well culture dishes at 1 × 10^3^ cells per well and cultured with corresponding material extracts (Xu et al., 2025). Experimental conditions mirrored those used in previous CCK-8 proliferation tests. After 24, 72, 120, and 168 h of culture, fluorescent viability indicators were administered according to standardized protocols. Cellular viability was quantified using an inverted fluorescence imaging system (DMi8, Leica, Germany), with viable cells fluorescing green and nonviable cells exhibiting red fluorescence. Parallel cultures maintained in standard growth medium functioned as experimental controls.

### 4.4 *In vivo* animal studies

#### 4.4.1 Surgical procedures

Female New Zealand albino rabbits (2.0–2.5 kg body weight) were obtained from the Laboratory Animal Research Facility at Xinjiang Medical University (Urumqi, China). All experimental procedures followed international standards for animal research as outlined in the ARVO Statement and were approved by the Ethics Review Board at the First Affiliated Hospital of Xinjiang Medical University (Ethics Approval Code: IACUC-JT-20231010-40). A total of 26 New Zealand white rabbits were used in this study and were randomly assigned to three groups: a blank control group (n = 6,healthy animals with no surgical intervention), a GelMA/HA-NB/LAP hydrogel filling group (n = 10), and a combined GelMA/HA-NB/LAP hydrogel and lenticule filling group (n = 10). All surgical procedures were performed exclusively on the right eye of each rabbit to avoid potential interocular interference. Surgical interventions employed combined anesthesia protocols consisting of xylazine combined with Zoletil 50 (1:1 mixture, administered intramuscularly at 0.15 mL/kg) augmented with topical application of 0.5% proparacaine hydrochloride eye drops for ocular surface anesthesia (Zhao X. et al., 2022). The operative procedure involved using a 7 mm diameter trephine to perform central corneal partial-thickness incisions on recipient eyes, followed by sequential stromal layer separation extending to approximately 50% of the corneal depth through meticulous manual dissection.

In the GelMA/HA-NB/LAP with lenticule transplantation group, two donor lenticules measuring 6.5–6.9 mm in diameter and 100–140 μm thickness were processed as graft materials (Jazayeri et al., 2025). The prepared lenticules were transplanted onto the recipient stromal interface and carefully aligned. The prehydrogel solution (GelMA/HA-NB/LAP) was applied. This solution exhibited moderate viscosity, facilitating easy injection and uniform spreading. After being applied to the corneal defect area, the solution adhered effectively to the wound bed without noticeable overflow. Following the removal of excess liquid with a sponge, the gel maintained stable positioning, indicating its favorable intraoperative manipulability and retention capability. Crosslinking was achieved through ultraviolet irradiation (365 nm, 12 W) maintained for 300 s.

For the hydrogel-only control group (GelMA/HA-NB/LAP), the stromal bed underwent prehydrogel infusion followed by meticulous surface hydration control using absorbent sponges prior to identical UV-induced polymerization parameters (365 nm, 12 W, 5 min) (Zhao X. et al., 2022).

The average operative time from incision preparation to the completion of photo-crosslinking was approximately 15 min, featuring a simple and efficient operational procedure. All operations were performed by the same senior ophthalmologist, and no complications including material slippage or severe hemorrhage occurred intraoperatively.

Postoperative management protocol included prophylactic administration of 0.5% levofloxacin ophthalmic solution combined with tobramycin/dexamethasone eye drops, maintained through three daily applications for 28 consecutive days.

#### 4.4.2 Clinical assessment

Clinical evaluation of corneal wound healing progression was systematically monitored across a 35-day observation window. Postoperative assessments included slit-lamp examinations (Topcon, Japan) and anterior segment optical coherence tomography (ZW-30, Towardpi, Beijing) conducted on days 1, 14, 21, 28, and 35 following surgery. Corneal structural analysis was complemented by Pentacam topography (Oculus, Germany) during the final evaluation week. The slit-lamp methodology enabled detailed observation of wound closure integrity, tissue clarity, inflammatory responses, vascular proliferation, and other morphological alterations. Epithelial defects were quantified through sodium fluorescein application techniques (Wang et al., 2024). AS-OCT imaging provided cross-sectional visualization of wound edge approximation and hydrogel dissolution patterns. Three-dimensional curvature analysis was performed using the Pentacam system to document corneal surface remodeling.

#### 4.4.3 Tissue HE staining

At 5 weeks post-surgery, rabbits received humane euthanasia through intravenous injection of pentobarbital sodium solution (10% w/v) delivered via the marginal auricular vein. Ocular specimens were carefully excised for histopathological analysis, undergoing sequential processing including fixation in 4% paraformaldehyde (PFA), paraffin embedding, and hematoxylin-eosin (HE) staining. Tissue sections were digitally analyzed using a 3Dhistech Pannoramic MIDI digital slide scanner, with high-resolution corneal images captured to evaluate potential interfacial discontinuities between implant and host tissue.

#### 4.4.4 SEM evaluation of tissues

Corneal specimens were obtained and analyzed through scanning electron microscopy (SEM; JSM-7610FPlus, JEOL, Japan) to detect the existence of visible discontinuities at graft-host junctions. Briefly, samples underwent fixation with 2.5% glutaraldehyde in darkness at 4 °C, followed by three PBS rinses lasting 15 min each. Ethanol gradient dehydration was subsequently implemented through sequential immersion in 30%–95% ethanol solutions (15 min per concentration). Complete dehydration was achieved through two 20-min treatments with absolute ethanol.

## 5 Statistical analysis

All experiments were independently repeated at least three times under standardized conditions. Data are presented as mean ± SD. Statistical analysis was performed using GraphPad Prism 10 software (GraphPad Software, United States). Comparisons between two subgroups within the same group were made using the paired Student’s t-test, with a P < 0.05 considered statistically significant.

## Data Availability

The original contributions presented in the study are included in the article/supplementary material, further inquiries can be directed to the corresponding authors.
